# Seeds of native alpine plants host unique microbial communities embedded in cross-kingdom networks

**DOI:** 10.1186/s40168-019-0723-5

**Published:** 2019-07-24

**Authors:** Birgit Wassermann, Tomislav Cernava, Henry Müller, Christian Berg, Gabriele Berg

**Affiliations:** 1 Institute of Environmental Biotechnology, Graz University of Technology, Petersgasse 12, 8010 Graz, Austria; 2 Institute of Biology, Department of Plant Sciences, NAWI Graz, University of Graz, 8010 Graz, Austria

**Keywords:** Seed microbiota, native plants, cross-kingdom networks, endophytes, plant resilience

## Abstract

**Background:**

The plant microbiota is crucial for plant health and growth. Recently, vertical transmission of a beneficial core microbiota was identified for crop seeds, but for native plants, complementary mechanisms are almost completely unknown.

**Methods:**

We studied the seeds of eight native plant species growing together for centuries under the same environmental conditions in Alpine meadows (Austria) by qPCR, FISH-CLSM, and amplicon sequencing targeting bacteria, archaea, and fungi.

**Results:**

Bacteria and fungi were determined with approx. 10^10^ gene copy numbers g^−1^ seed as abundant inhabitants. Archaea, which were newly discovered as seed endophytes, are less and represent only 1.1% of the signatures. The seed microbiome was highly diversified, and all seeds showed a species-specific, highly unique microbial signature, sharing an exceptionally small core microbiome. The plant genotype (species) was clearly identified as the main driver, while different life cycles (annual/perennial) had less impact on the microbiota composition, and fruit morphology (capsule/achene) had no significant impact. A network analysis revealed significant co-occurrence patterns for bacteria and archaea, contrasting with an independent fungal network that was dominated by mutual exclusions.

**Conclusions:**

These novel insights into the native seed microbiome contribute to a deeper understanding of seed microbial diversity and phytopathological processes for plant health, and beyond that for ecosystem plasticity and diversification within plant-specific microbiota.

**Electronic supplementary material:**

The online version of this article (10.1186/s40168-019-0723-5) contains supplementary material, which is available to authorized users.

## Background

Plants and their associated microbes have been interacting with each other for a long time, forming an assemblage of species that is often referred to as a holobiont [1]. The plant microbiome is essential for plant development, resilience, and health [2]. Endophytic communities represent an intimate core of the plant microbiota and connect different plant microhabitats, with specific roles during development stages that are important for health issues [3]. The rhizosphere is a reservoir for plant endophytes and represents the below-ground interface with the highly diverse soil microbiota [4]. For a long time, it was assumed that the emerging seedling is colonized by microbes from its surrounding environment, with soil being the main source and plant-controlled enrichment through different strategies, such as the specific profile of root exudates and its immune system [5–7]. Therefore, studies related to the seed microbiota have often been neglected or focused only on the presence of pathogens [8]; there are less comprehensive studies including all components of the microbiome [9]. In the past, only a few studies on seed-derived bacteria were published, because they are difficult to cultivate, while seed-borne archaea were not identified at all so far [10, 11]. Our knowledge related to seed fungi is much broader as reviewed by Geisen et al. [12] and Yang et al. [13], especially in terms of information available on clavicipitaceous endophytes such as *Epichloë/Neotyphodium* species due to their beneficial and specific interaction with grasses, which is already commercially exploited [14, 15]. However, we still need a complete picture of the seed microbiota and its interactions and functions in the holobiont.

Recently, crop seeds were discovered as a source to transmit a plant-specific core microbiota [16–18]. Studies focusing on the crop microbiome showed that domestication and intense agricultural management entailed alterations of the inherent microbiome of crop plants including a loss of plant-beneficial microbiota [19–21]. First results indicate similar effects on the seed microbiota [16, 22]. Understanding the key components of the indigenous seed microbiota of native plants can support the definition of a healthy microbiota and its translation to our crops. So far, little is known about the indigenous seed microbiota in natural ecosystems [12]. Our hypothesis was that seeds of native plants harbor a specific and diverse microbiota, which allows plant populations to survive, persist, and germinate under harsh natural conditions [23, 24].

To decipher the entire seed microbiota of native plants, we selected healthy plant populations persisting over centuries in the European Eastern Alps. The Hochschwab region (Northern Calcareous Alps, Austria) is a glacial retreat for a high diversity of plant species and is botanically and geologically well-studied [25]. To identify the composition and main drivers (plant genotype, life cycle, fruit morphology) of the native seed microbiota, we selected eight different alpine plant species, all of which were traditionally used as medicinal plants and produce a variety of antimicrobial compounds [26, 27]. Although nothing is known about bioactive compounds in the seeds of the selected species, we expect that those phytochemicals have profound impacts on the seed microbiota. In addition, we hypothesize a strong impact of fruit morphology; here, we expected a higher microbial diversity for seeds of indehiscent fruits (achenes) than for seeds of dehiscent fruits (capsules). Achenes are monocarpellate structures, where the seeds are united with the pericarp forming a unit developed and distributed under the influence of the surrounding environment. A separation between seeds and fruit in achenes is not possible methodically, and we use the term seeds in the following text including achenes. Capsules are enclosed systems, where seeds develop inside, covered by the pericarp that splits apart to extrude the seeds at maturity. In these structures, the surrounding environment has a lower impact on the seed microbiome. The selected plants are also characterized by a different life cycle (annuals/perennials). We hypothesize that perennials can accumulate a higher microbial diversity during their life cycle.

## Materials and methods

### Experimental design and sampling procedure

**Fig. 1:**
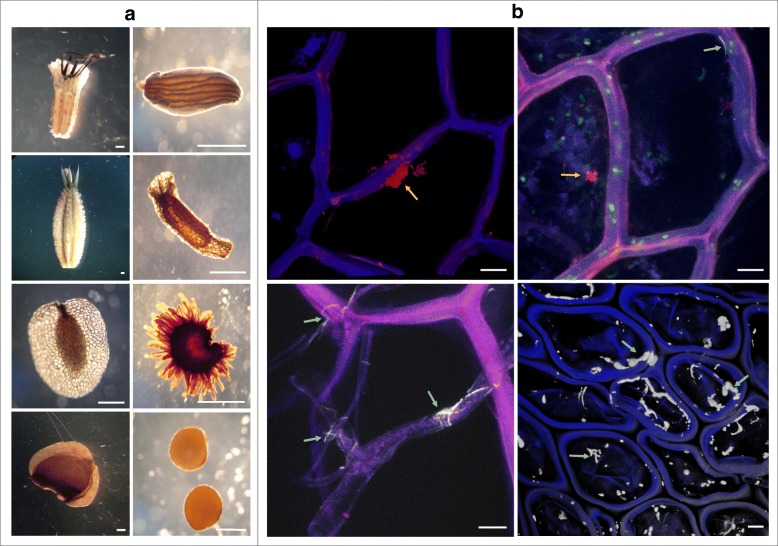
Micrographs of alpine plant seeds (including achenes). **a** Whole seed visualization shows the high morphological diversity of the alpine plant seeds investigated, from left to right and top to bottom: *S. lucida*, *E. rostkoviana*, *A. major*, *P. palustris*, *G. asclepiadea*, *H. quadrifida*, *R. glacialis*, and *G. germanica*. Scale bars in **a** indicate 0.5 mm. **b** FISH-Confocal laser scanning micrographs visualize endophytic fungi (indicated by *green arrows*) and bacteria (*yellow arrows*) in *P. palustris* and *G. asclepiadea* seeds. Scale bars in **b** indicate 10 μm

For the microbiome analyses, eight different alpine plant species (in the following referred to as plant genotypes) were selected according to different life cycles and fruit morphologies. Our selection comprised the following species: great masterwort *Astrantia major* L., Eyebright *Euphrasia rostkoviana* Hayne, willow gentian *Gentiana asclepiadea* L*.*, Chiltern gentian *Gentianella germanica* (Willd.) E.F.Warb*.*, *Heliosperma quadrifida* Waldst. & Kit., bog star *Parnassia palustris* L., Yellow rattle *Rhinanthus glacialis* Personnat, and pincushion flowers *Scabiosa lucida* Vill. These plant species differ in their fruit morphologies; *E. rostkoviana*, *R. glacialis*, *G. germanica*, *H. quadrifida*, *P. palustris*, and *G. asclepiadea* produce capsules as dehiscent fruits and *S. lucida* and *A. major* seeds produce achenes as indehiscent fruits. Plants can furthermore be distinguished by their life cycle, which is either annual (*E. rostkoviana*, *R. glacialis*, and *G. germanica*) or perennial (*H. quadrifida*, *P. palustris*, *G. asclepiadea*, *S. lucida*, and *A. major*). All seeds were collected at time of dispersal in maturation state. The sampling was performed on September 4, 2016, at the Aflenzer Staritzen (Longitude: E15.183899, Latitude: N47.622001) in an area of approximately 100,000 m^2^ in the Hochschwab region (Northern Calcareous Alps, Austria), which represent a botanically well-studied glacial retreat [25]. Each of the eight plant species was sampled from four different sites randomly selected across the total area. Each replicate consists of 15 to 20 plants that grew in close proximity (subpopulations). The distance between the replicates was 200 m in minimum. Seeds of plants from one subpopulation were handled under sterile conditions and subsequently pooled. From each pool, 50 mg were weighted in, now referred to as one replicate, and total community DNA was extracted. We decided to use consistent seed weights for each replicate instead of seed counts due to strong variability in seed size and anatomy between the different plant genotypes (Fig. 1a).

### Microbial DNA extraction and amplicon library construction

Seeds were physically disrupted under sterile conditions with liquid nitrogen, and the total community DNA was extracted using the FastDNA Spin Kit for Soil (MP Biomedicals, Solon, USA) and a FastPrep Instrument (MP Biomedicals, Illkirch, France) for 30 s at 5.0 ms^−1^. Illumina amplicon sequencing was performed by using two different barcoded primer combinations: 515f–806r [28] to amplify 16S rRNA gene fragments and ITS1f–ITS2r [29] to amplify parts of the ITS region, with three technical replicates per sample. By adding peptide nucleic acid (PNA) clamps to the PCR mix, amplification of host plastid and mitochondrial 16S DNA was blocked [30]. PCR for 16S rRNA gene amplification was performed in a total volume of 30 μl (5 x Taq&Go (MP Biomedicals, Illkirch, France), 1.5 μM PNA mix, 0.25 mM of each primer, PCR-grade water, and 1 μl template DNA) under the following cycling conditions: 95 °C for 5 min, 30 cycles of 96 °C for 1 min, 78 °C for 5 s, 54 °C for 1 min, 74 °C for 60 s, and a final elongation at 74 °C for 10 min. Amplification of the fungal ITS region was conducted in 20 μl (5 x Taq&Go, 25 mM MgCl_2_, 10 μM of each primer, PCR-grade water, and 1 μl template DNA) with the cycling conditions 95 °C for 5 min, 30 cycles of 94 °C for 30 s, 58 °C for 35 s, 72 °C for 40 s, and final elongation at 72 °C for 10 min. A nested PCR step was performed to add barcoded primers. Technical replicates were combined and purified by Wizard SV Gel and PCR Clean-Up System (Promega, Madison, WI, USA), and DNA concentrations were measured with Nanodrop 2000 (Thermo Scientific, Wilmington, DE, USA). Samples were combined in equimolar concentration and sequenced by Illumina MiSeq v2 (250 bp paired-end) amplicon sequencing.

### Illumina MiSeq data processing of 16S rRNA gene and ITS region amplicons and statistics

Raw sequence data preparation and data analysis was performed using QIIME 1.9.1 [28]. Paired reads were joined and quality filtered (phred q20), and chimeric sequences were identified using usearch7 [31] and removed. Operational taxonomic units (OTUs) were picked according to open references given by SILVA ver128_97_01.12.17 for 16S rRNA gene and UNITE ver7_99_01.12.17 for fungal ITS region. De novo clustering of OTUs was performed using usearch for bacterial and archaeal 16S rRNA and BLAST for fungal ITS region. Representative sequences were aligned, taxonomy was assigned, and sequences assigned to host mitochondria and chloroplasts were discarded. OTU tables were rarefied to the lowest number of read counts (1739 sequences for 16S rRNA gene and 5807 sequences for ITS region). Bacterial and fungal core OTUs that were present in all alpine plant seeds investigated were further identified up to species level using NCBI BLAST alignment tool. Rarefied OTU tables served as input matrix for all upcoming alpha and beta diversity analyses. Statistics on microbial diversity and abundance were calculated in QIIME. Significant differences (*p* < 0.05) in Shannon diversity between groups for 16S rRNA gene fragments and for the ITS region were calculated based on parametric two-sample *t* test at the greatest rarefaction depth using *t* distribution to determine the *p* value. Beta diversity, based on weighted UniFraq distance matrix for bacteria and Bray-Curtis dissimilarities for fungi, was assessed by principal coordinates analysis (PCoA), and the statistical significance between categorical variables was assessed by analysis of similarity (ANOSIM), including the pairwise option to compare differences between all plant genotypes. IBM SPSS program (version 25.0, IBM Corporation, Armonk, NY, USA) was used for calculating significant differences (*p* < 0.05) in microbial gene copy quantity, determined via quantitative PCR, based on ANOVA including Tukey-HSD test correction. Cytoscape version 3.4.0 and the add-on “CoNet” were used to perform network analysis of significant (*q* ≥ 0.0004) co-occurrence and mutual exclusion patterns of the microbiomes. Combined fungal and bacterial OTU table, collapsed on species level using absolute abundances, served as input matrix for the co-occurrence network. To ensemble inferences, Pearson and Spearman correlation coefficients for both positive and negative correlations, using the automatic threshold setting for the 1000 top and bottom edges for each method, the mutual information option, and Bray-Curtis and Kullback-Leibler dissimilarity matrices were applied. For the final network, bootstrapping was selected as resampling method and Brown’s method was used to merge method- and edge-specific *p* values, discarding unstable edges that showed scores outside the 0.95% range of their bootstrap distribution. The Benjamini-Hochberg method was selected for multiple test correction.

### Quantitative real-time PCR

For quantifying gene copy numbers of bacteria, archaea, and fungi within seeds, a quantitative real-time PCR (qPCR) was performed using the following primer pairs: 515f–927r for bacteria (10 μM each; [32]), 344aF–517uR for archaea (5 μM each; [33]), and ITS1–ITS2 for fungi (10 μM each; [29]). The reaction mix contained 5 μl KAPA SYBR Green, 0.5 μl of each primer, 3 μl PCR-grade water, and 1 μl template DNA (diluted 1:10 in PCR grade water). Fluorescence intensities were detected in a Rotor-Gene 6000 real-time rotary analyzer (Corbett Research, Sydney, Australia) with the following cycling conditions: bacteria: 95 °C for 5 min, 40 cycles of 95 °C for 20 s, 54 °C for 30 s, 72 °C for 30 s, and a final melt curve of 72 to 96 °C; archaea: 95 °C for 5 min, 40 cycles of 95 °C for 15 s, 60 °C for 30 s, 72 °C for 30 s, followed by melt curve of 72 to 96 °C; fungi: 95 °C for 5 min, 40 cycles of 95 °C for 30 s, 58 °C for 35 s, 72 °C for 40 s with a melt curve of 72 to 96 °C. Three individual qPCR runs were conducted for each replicate. Intermittently occurring gene copy numbers that were found in negative controls were subtracted from the respective sample.

### Fluorescent in situ hybridization and confocal laser scanning microscopy

In-tube fluorescent in situ hybridization (FISH) technique, followed by visualization with confocal laser scanning microscopy (CLSM), was performed to observe the colonization patterns and penetration spots of seed-associated bacteria and fungi. Seeds were fixed with 4% paraformaldehyde/phosphate-buffered saline at 4 °C over-night prior to FISH application according to the protocol of Cardinale et al. [34]. To stain the overall bacterial community, Cy3-labeled EUB338MIX [35] was used and in order to contrast fungal structures from plant cell walls; FISH samples were treated with Calcofluor White.

## Results

### Visualization of microbial communities and their abundance in alpine plant seeds

Seed morphology and size of the eight alpine plants investigated were highly variable, specifically adapted to their mode of dispersal. Seed size ranged from 0.7 mm for *G. germanica* seeds up to 6.5 mm for *A. major* seeds (Fig. 1a). We used different observation methods to explore microbial colonization patterns on seeds. Scanning electron microscopy was applied to monitor seed surfaces for microbial colonization; here, only few epiphytes were detected. Among them, fungal structures were more frequent than bacterial ones (results not shown). In addition, CLSM in combination with specific FISH probes allows to localize endophytes in different sub-compartments of the seeds. Visualization was feasible for *P. palustris* and *G. asclepiadea* seeds (Fig. 1b). We found fungal structures more frequently than bacterial ones, and especially, the surface of *P. palustris* was covered with fungal hyphae. Comparably less Cy3-labeled bacteria were visualized colonizing seeds epi- and endophytically. Unfortunately, high autofluorescence of host tissues impeded imaging of microbiota in the seeds of the remaining plant genotypes.

**Fig. 2:**
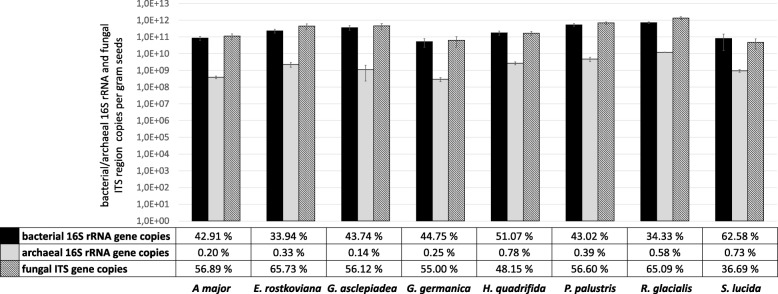
Microbial gene copy numbers in alpine plant seeds determined by qPCR. Values are given by primers targeting bacterial and archaeal 16S rRNA gene and fungal ITS region in seeds of the eight different alpine plants. Gene copy numbers are calculated per gram seeds used for the microbiome analysis. The table describes the prokaryote to eukaryote ratio within the total microbial gene copies detected in the seeds of the respective plant genotype. Total microbial gene copies can be looked up in Additional file 1: Table S1

In contrast, quantification via qPCR resulted in high microbial abundances in all seeds investigated, amounting for 2.8 × 10^11^, 3.09 × 10^9^, and 4.2 × 10^11^ mean gene copy numbers per gram seeds for bacteria, archaea, and fungi, respectively (Fig. 2). Significant differences in microbial abundance were observed between the eight plant genotypes, whereas comparing fruit morphology (capsule or achene) or life cycle of the plant (annual or perennial) resulted in no statistical significance (Additional file 1: Table S1). This holds true both for the number of total microbial gene copies and for bacteria, archaea, and fungi calculated separately. The total microbial gene copies per plant genotype, consisting of bacteria, archaea, and fungi, ranged from 1.16 × 10^11^ gene copies in *G. germanica* seeds to 2.10 × 10^12^ gene copies in *R. glacialis* seeds. However, calculating the prokaryote to eukaryote ratio, indicated by percent values in Fig. 2, resulted in high similarities between the different plant genotypes. Fungal ITS gene copies slightly prevailed over bacterial and archaeal 16S rRNA gene copies, except for *H. quadrifida* and *S. lucida* seeds. Archaeal gene copies were detected in all replicates; however, less than 1% out of total microbial genes per plant genotype were archaeal. This ratio was found to be consistent over the sample collection, and no mutual exclusions between the three taxonomic groups were observed: seeds with high bacterial gene copies (*R. glacialis*, *P. palustris*, *G. asclepiadea*, and *E. rostkoviana*) showed high copy numbers of archaeal and fungal genes as well, while seeds with less bacterial copy numbers (*A. major*, *G. germanica*, and *S. lucida*) exhibit also less archaeal and fungal gene copies.

With respect to both the microscopic and the quantitative evaluations, the majority of seed-associated microorganisms is most probably localized inside the seeds.

### Unique microbial composition associated within alpine plant seeds

After quality-filtering and removing chimeric sequences, the amplicon dataset with the 16S rRNA gene fragments from alpine plant seeds contained 4,703,620 paired reads. Chloroplast and mitochondrial sequences were removed, and 1,769,793 sequences remained in the dataset that were assigned to a total of 11,842 operational taxonomic units (OTUs). The quality-filtered and chimera-checked ITS dataset contained 10,443,899 paired reads that were assigned to 3943 fungal OTUs. OTU tables were rarefied to 1739 bacterial and archaeal sequences and 5807 fungal sequences per sample, according to the samples with lowest amount of sequences.

The taxonomic assignment of 11,844 bacterial OTUs revealed 36 phyla, among them *Proteobacteria* were predominant in the dataset with 88.9%, followed by *Actinobacteria* (3.3%) and *Bacteroidetes* (2.2%). *γ-Proteobacteria* was the most abundant class received, amounting to 48.7% relative abundance and represented by 83 genera. *α-Proteobacteria* showed the highest diversity, being represented by 395 genera and amounting to 34.4% relative abundance. *Actinobacteria* and *Bacteroidetes* were represented by 200 and 68 genera, respectively. *Firmicutes* (141 genera), *Acidobacteria* (51 genera), *Chloroflexi* (45 genera), *Planctomycetes* (38 genera), *Verrucomicrobia* (26 genera), and *Cyanobacteria* (21 genera) showed each less than 1% abundance in the whole dataset. The remaining and less represented taxa are not described here.

Archaeal taxonomy was assigned to 32 OTUs and represented 0.05% of the 16S rRNA gene sequences. Archaea were mainly represented by *Thaumarchaeota* (98.3% rel. abundance of all archaeal sequences) with three genera of the Soil Crenarchaeotic Group and *Nitrosphaera*. *Euryarchaeota* were less represented, amounting to 1.7% of archaeal community but were more diverse, consisting of the genera *Haladaptatus*, *Methanobacterium*, *Methanobrevibacter*, *Natronorubrum*, *Methanosphaera*, and one not further assigned genus of *Halobacteriaceae*.

The ITS amplicon library was assigned to 3945 fungal OTUs. Most abundant OTUs included *Ascomycota* with 74.2% relative abundance and 274 genera and *Basidiomycota* with 25.8% abundance and 119 genera. Among *Ascomycota*, the majority of OTUs were assigned to *Dothideomycetes* (50.5% abundance and 93 genera), *Sordariomycetes* (1.3% and 72 genera), *Leotiomycetes* (5.9% and 48 genera), and *Eurotiomycetes* (2.1% and 16 genera). The most abundant class within *Basidiomycota* was *Tremellomycetes* (23.0%), although poorly diverse with only 19 genera. *Microbotryomycetes* (2.3% and 11 genera) and *Agaricomycetes* (0.3% and 77 genera) represented the remaining *Basidiomycota*. *Chytridiomycota* (0.04% and three genera) and *Glomeromycota* (0.002%), *Rozellomycota* (0.001%) and *Zygomycota* (0.0005%), each represented by one genus, were much less abundant.

The composition of bacterial, archaeal, and fungal genera that were present in each replicate of a sample is visualized in Additional file 1: Figure S1. For bacteria and fungi, a threshold of 0.01% abundance was set. As most archaea were present with less than 0.01% in the 16S rRNA gene library, no threshold was set for archaeal composition to be included in Additional file 2: Figure S2. Those highly diversified microbiomes included 70 bacterial, 10 archaeal, and 58 fugal genera. Among them, some highly abundant genera were shared between the seed core microbiomes of all plant genotypes, but the relative abundance of those showed high variations. *Sphingomonas*, *Pseudomonas*, *Tatumella*, or *Pantoea*, known for their ubiquitous association with plants, were present in all seed core microbiomes (Additional file 1: Figure S1A); the same was true for three different, but not further assigned archaeal taxa of Soil Crenarchaeotic Group (Additional file 1: Figure S1B). Fungal composition showed some consistencies among high abundant *Cryptococcus*, *Cladosporium*, or *Davidiella* (Additional file 1: Figure S1C). The mean relative abundance of all bacteria and archaea and all fungi in the whole dataset with at least 0.01% abundance are listed in Additional file 1: Table S2 and S3, respectively.

### Identification of the main drivers of the native seed microbiome

The bacterial and fungal diversity within the alpine plant was assessed by Shannon diversity index, and significant differences (*p* < 0.05) between plant genotypes were calculated using the parametric two-sample *t* test at the greatest rarefaction depth. The samples were either grouped by plant genotype, the life cycle, or the fruit type, in order to identify dependencies of microbial diversity on either category (Additional file 2: Figure S2 and Table S4). When the samples were grouped by their plant genotype, values for bacterial diversity ranged from *E. rostkoviana* seeds (5.09) to *R. glacialis* seeds (2.4). Diversity of *E. rostkoviana* seed microbiome was found to be significantly higher than *R. glacialis* and *G. germanica* seed microbiomes. Significant differences in fungal diversity were observed between most diverse *G. asclepiadea* seeds (5.09) and *R.* glacialis, *P. palustris*, *G. germanica*, *E. rostkoviana*, and *H. quadrifida* seeds. *H. quadrifida* seed mycobiome was furthermore significantly more diverse than the one of *G. germanica*. *A. major*’s seed mycobiome was significantly more diverse than *R. glacialis*’. The mycobiome diversity is therefore suggested to be more dependent on the plant genotype than the bacterial diversity. When the samples were grouped by their life cycle, no significant differences in diversity were observed for the bacterial microbiota, while the mycobiome of perennial plant seeds (4.53 ± 0.05) was significantly more diverse than the mycobiome (3.12 ± 0.05) of annuals. No significant difference in Shannon diversity for both bacteria and fungi was observed when the samples were grouped by their fruit type.

**Fig. 3:**
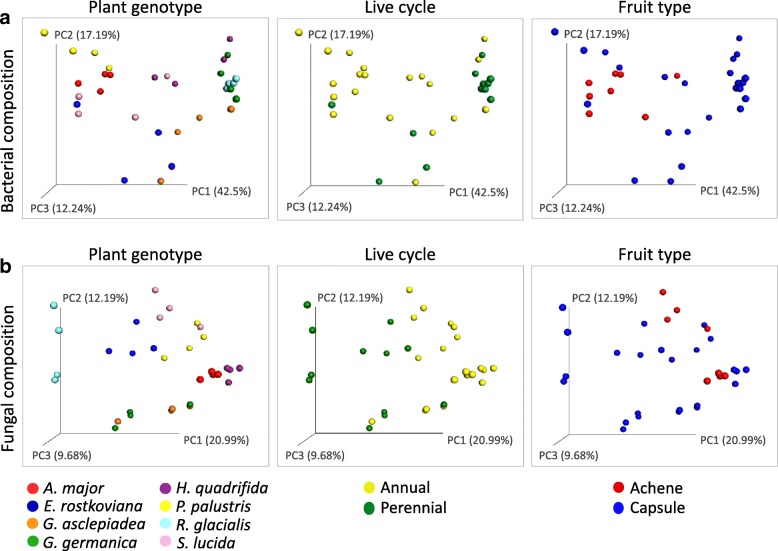
Beta-diversity analysis calculating microbiome composition dependencies on either categorical variable. PCoA plots are based on weighted UniFraq distance matrix for bacterial community (**a**) and on Bray-Curtis dissimilarities for the fungal community (**b**) of seed microbiomes. Bacterial and fungal composition of the samples are grouped by plant genotype, plant’s life cycle, and the fruit type. Color codes are explained in the legends below

In order to evaluate the main driver of the seed microbiome composition, beta-diversity analysis was conducted using PCoA (Fig. 3) in combination with ANOSIM (Additional file 2: Table S5). Among the selected categorical variables “plant genotype,” “life cycle,” and “fruit type,” the plant genotype was found be the main driver of the microbial composition of alpine plant seeds. This applies both for bacteria (*R* = 0.509; *p* = 0.001) and fungi (*R* = 0.612; *p* = 0.001). The bacterial composition seems to be further dependent on the plant’s life cycle (*R* = 0.198; *p* = 0.004), either annual or perennial, while the life cycle dependency was even higher for fungi (*R* = 0.395; *p* = 0.001). The fruit type (capsule or achene) had no impact on the microbial composition giving the following ANOSIM values: *R* = 0.058; *p* = 0.23 for bacteria and *R* = − 0.029; *p* = 0.584 for fungi. The ANOSIM pairwise option was applied to compare the seed microbiomes of all plant genotypes; among the 28 combinations, 18 and 22 were significantly different for the bacterial and the fungal community, respectively (Additional file 2: Table S6). These results indicate that the fungal community has a higher plant genotype specificity than the bacterial community. However, it cannot be argued that two plant genotypes harbor similar microbial communities, as for all combinations either the bacterial or the fungal microbiome was significantly different.

### The limited seed core microbiome validates the unique signature of the plant genotype

In order to evaluate the dissimilarities between the seed microbiomes of the eight plant genotypes, the amount of shared OTUs was quantified. Only eleven out of 11,810 bacterial OTUs and only five out of 3945 fungal OTUs were present in all seeds (Table 1). This amounts to a percentage of 0.09% shared bacterial OTUs and 0.13% shared fungal OTUs, assigning bacteria a slight, but even higher plant genotype-dependent composition than fungi. Those bacterial and fungal OTUs represented the exceedingly undersized core microbiome shared by all alpine plant seeds. Regarding archaea, not a single OTU out of 32 OTUs was shared. A genotype dependency of archaea is therefore highly assumed as well; however, a number of 32 OTUs is too low to give a clear assessment. The abundances of the shared bacterial and fungal OTUs varied considerably between the samples; hence, these shared OTUs are not coincidently highly abundant in all seeds.

**Table 1:** Abundance of core OTUs in all seeds and percentage of OTUs exclusive for the core microbiomes of each plant genotype

		*A. major*	*E. rostkoviana*	*G. asclepiadea*	*G. germanica*	*H. quadrifida*	*P. palustris*	*R. glacialis*	*S. lucida*
Bacterial core OTUs shared by all seeds*	*Tatumella sp.*	0.21	1.94	6.06	24.60	0.95	0.69	75.84	2.42
	*Pseudomonas sp.*	13.84	5.39	29.51	0.32	32.27	5.40	0.06	7.63
	*Pseudomonas putida*	0.17	0.26	1.73	27.40	9.48	0.14	0.24	0.65
	*Pseudomonas fluorescens*	0.41	0.47	2.02	0.49	12.43	0.34	0.62	2.64
	*Serratia liquefaciens*	0.75	1.15	0.77	0.37	1.11	0.72	1.35	4.17
	*Burkholderia sp.*	0.16	0.28	0.30	0.10	0.35	0.34	0.73	2.80
	*Bacillus subtilis*	0.20	0.19	0.23	0.08	0.34	0.28	0.70	0.13
	*Pseudomonas protegens*	0.13	0.26	0.16	0.17	0.62	0.08	0.12	0.21
	*Curvibacter gracilis*	0.05	0.07	0.09	0.03	0.14	0.10	0.17	0.61
	*Pelomonas sp.*	0.02	0.04	0.04	0.01	0.06	0.05	0.15	0.64
	*Acinetobacter calcoaceticus*	0.04	0.07	0.03	0.02	0.05	0.04	0.05	0.09
Percentage of bacterial OTUs unique for the plant´s core microbiome**		44.1	65.7	38.0	31.3	11.4	21.7	15.4	5.2
Fungal core OTUs shared by all seeds*	*Cladosporium cladosporioides*	7.31	27.37	14.55	46.33	2.41	3.59	25.77	6.53
	*Cryptococcus victoriae*	15.14	1.78	8.05	4.64	28.21	14.34	0.02	8.03
	*Davidiella tassiana*	6.40	7.14	14.79	15.52	12.34	14.54	1.18	5.07
	*Boeremia exigua var. populi*	0.46	10.95	3.11	0.14	1.98	29.00	0.03	19.77
	*Epicoccum nigrum*	4.94	8.76	1.45	2.20	6.53	1.16	1.57	11.40
Percentage of fungal OTUs unique for the plant’s core microbiome**		33.1	35.7	76.1	12.5	31.0	12.7	23.5	26.5

*Numbers denote for relative abundance (%) in seeds of each plant genotype. Taxonomy was assigned at species level to core OTUs using NCBI BLAST alignment tool

**Percentage of OTUs occurring in all replicates of the respective plant species, while being absent in the core microbiomes of all other plant species

The percentage amount of OTUs occurring exclusively in the core microbiomes of either plant genotype, while being absent in the core microbiomes of the other plants, was furthermore assessed (Table 1). Here, the core microbiome refers to OTUs that are present in all replicates of a plant species. The calculation revealed a highly specific seed microbiome for each plant genotype, reaching from 65.7% unique OTUs in *E. rostkoviana* seeds to 5.2% unique OTUs in *S. lucida* seeds. Unique fungal OTUs per plant genotype were even more frequent, reaching from 76.1% unique OTUs in *G. asclepiadea* seeds to 12.7% unique OTUs in *P. palustris* seeds. Those results further promote the clear plant genotype dependency of the seed microbiomes.

### Contrasting interconnections of bacteria, archaea, and fungi within the microbial network

**Fig. 4:**
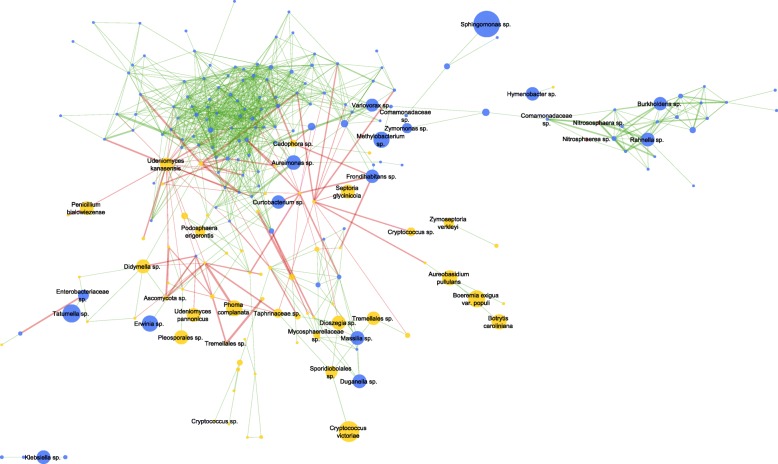
Co-occurrence and mutual exclusion relationships among seed-associated microbiota of alpine plants. Network was prepared by combining taxonomic assignment to OTUs on species level of 16S rRNA and ITS amplicon dataset. Only significant interactions are shown (*q* ≥ 0.0004). Color of nodes represent the three taxonomic groups (*blue*: bacteria, *yellow*: fungi, *pink*: archaea), and the size of nodes is proportional to the abundance of the taxon. Color of edges indicates the type of the interaction (*green*: positive or co-occurrence, *red*: negative or mutual exclusion), and the edge width is proportional to the significance. Taxonomy for high-abundant taxa is included

In order to illustrate general co-occurrence patterns of the seed microbiota across all plant genotypes, a network analysis was performed (Fig. 4). The network, showing significant co-occurrence and mutual exclusion patterns of the seed-associated microbiota, consists of 223 nodes, a characteristic path length of 4.392, and a network density with 0.044. The vast majority of bacteria represent a very dense and highly interactive part of the network where exclusively positive interactions occur. The remaining bacteria, partially distantly located to this dense part, show only positive interactions as well. Archaea from the genus *Nitrososphaera* form a distinct and positive interacting cluster with some bacteria that are described for plant-beneficial properties. This distinct cluster is connected to the main network by a *Comamonadaceae* taxon. The entirety of negative interactions, i.e., mutual exclusions, was observed for fungi, located outside of the dense part of the bacterial network.

## Discussion

The results of this study confirm our hypothesis that seeds of native plants harbor a more specific microbiota than already discovered for crop plants. The key findings of this study were (i) the unexpected high microbial abundances mainly driven by the plant genotype, (ii) the consistent eukaryote to prokaryote ratio across all seeds investigated, (iii) the high degree of plant specificity shown for the entire microbiome, (iv) the first identification of plant-specific seed-borne archaea, (v) an exceptionally small core microbiome although all plants grow together for centuries in the same soil and under the same environmental conditions, and (vi) the network of bacteria and archaea, which was in contrast to the negatively interacting fungal network.

The alpine plants studied showed a unique degree of plant specificity compared to the present literature [36–38]. Differences between seed microbiomes of the plant genotypes were found in terms of bacterial and fungal composition, abundance, and diversity. Inter-kingdom symbiosis was genotype-specific as well, as seeds with high bacterial gene copy numbers also showed high copy numbers of archaea and fungi and vice versa. The seeds share an exceedingly undersized core microbiome where only 11 out of 11,810 bacterial OTUs, only five out of 3945 fungal OTUs, and not a single OTU out of 32 archaeal OTUs were shared. The five fungal OTUs, which form the core, are classified as black fungi [39] able to persist ecological harshness and even to convert radiation into metabolic activity [40]. Rezki et al. [41] recently described an even higher degree of individual-specific microbiota for radish seeds; only three bacterial and 19 fungal OTUs were shared. However, those OTUs covered 70% and 87% of all bacterial and fungal reads within the individual plants. Reduced diversity and low evenness might therefore be suggested for crop plants, in comparison with the alpine plants. Here, the core OTUs represented an average of 3% and 10% of all bacterial and fungal reads, respectively. The amount of OTUs specific for each plant genotype was furthermore substantial, reaching up to 65.7% for bacteria in *E. rostkoviana* seeds and 76.1% for fungi in *G. asclepiadea* seeds. However, direct comparison to [41] is limited as *gyrB* instead of 16S rRNA was selected as bacterial marker gene. Seeds of *R. glacialis* were found most unique, probably caused by specific, antimicrobial secondary metabolites [42]. Their microbial diversity was significantly lower, but the abundance was significantly higher compared to the other samples. In addition to the plant genotype, plant’s life cycle was found to have an impact on the microbiome composition, where seed microbiota of perennial plants differed significantly from seeds of annual plants. This can be explained by the possibility to accumulate microbial diversity protecting the plants also during winter time. By selecting plants with two different fruit types, capsules and achenes, we aimed to specify the influence of the surrounding environment on seed microbiomes, which was found surprisingly to be not significant. This indicates that the airborne contribution to the seed microbiome is negligible. Significant genotype and life cycle dependency of the seed-associated microbiota was obvious, although we decided not to distinguish between seed endo- and epiphytes and considered both as an entire community of the alpine plant seeds. However, our microscopic observations showed that seed surfaces were not or less colonized by microbes. Therefore, we promote the recent suggestion by Nelson [8] that the epiphytic seed community is less insignificant than previously expected.

According to present literature, a plant-pathogenic potential can be assigned to the majority of highly abundant seed-associated fungi (e.g., *Botrytis*, *Alternaria*, *Phoma*, *Didymella*, *Davidiella* [43–46]) while the abundance of fungal taxa with described parasitism towards other fungi was high as well (e.g., *Cryptococcus*, *Dioszegia* [47]). However, all seeds were sampled from healthy plant populations, persisting under the given conditions for many growing seasons; germination ability is therefore highly assumed. Resilience towards emerging pathogens and adverse environmental conditions is probably supported by a highly abundant and competitive mycobiome. The inter-kingdom co-occurrence network illustrates the antagonistic features of the mycobiome; exclusively all mutual exclusions were observed for fungi. This stands in vast contrast to the positively interacting bacterial network, indicating synergism and stability. Competition for resources and space within the seed mycobiome has already been proposed by Rezki et al. [48], where the invasion of a fungal plant pathogen altered the fungal, but not the bacterial seed community, and Johnston-Monje and Raizada [49] suggested stability of bacteria, based on conserved patterns of bacterial endophytes in *Zea* seeds across boundaries of evolution. Among archaea, two OTUs of *Nitrososphaera* were present in the significant inter-kingdom network, positively interacting with bacteria. Beyond that, archaea were detected in all seeds investigated. We therefore assume an important ecological function of this domain for plant health and development. The performance of co-occurrence networks can be interfered by the input matrix when metacommunities from different habitats are combined, which in such cases can result in co-occurrence due to the habitat sampled rather than direct biological interactions (habitat filtering effect) [50]. Simulation models and algorithms described in literature [50, 51] can remedy that issue, which however would require a greater sample size than the available in the present study. The sampling for the present study was conditioned by the number of plants per genotype grown on the alpine meadow. However, the sample size was still sufficient for the present network where statistical tests, reported to give highest specificity and sensitivity, were applied [51]. Interpretability of the present network inferring putative microbial interactions in alpine plant seeds is therefore feasible when keeping potential interferences in mind. Altogether, our results indicate specified functions within the whole microbial network: bacteria and archaea strengthen the beneficial interplay within the holobiont, while fungi are responsible for degradation of the organic matter, e.g., seed shell, and may be to condition and train the prokaryotic microbiome through their antagonistic pressure.

A comparison of the microbiota of native and crop seeds confirmed our hypothesis that seeds of native plants harbor a more specific and differentially composed microbiota in comparison with cultivated plants that were investigated so far. Links et al. [52] compared seed microbiomes of *Brassica* and *Triticum* crops and found a hundredfold higher amount of shared OTUs (578 out of 5477 OTUs) compared to alpine seeds. The contrast is increased by the fact that *Brassica* and *Triticum* seeds were originated from different locations, but showed still higher similarity than the seeds of alpine plants, sampled on less than 20,000 m^2^. Truyens et al. [5] reviewed that *Bacillus*, *Pseudomonas*, *Paenibacillus*, *Micrococcus*, *Staphylococcus*, *Pantoea*, and *Acinetobacter*, in ascending order, are the most common bacteria within seeds of very different crop species. This is only partially consistent with our results. Alpine plant seeds are dominated by *Pseudomonas*, *Sphingomonas*, *Tatumella*, *Methylobacterium*, and *Pantoea*. The abundance of *Bacillus*, *Acinetobacter*, and especially *Paenibacillus*, *Staphylococcus*, and *Micrococcus* was very low. Differences to comparable studies on crop seeds [17, 22, 49, 52–54] were also observed on higher taxonomic levels: alpine plant seeds showed higher abundance of *α-Proteobacteria* but far lower abundance of *Actinobacteria* and *Firmicutes*.

Until now, mainly crop seeds were studied. What can we learn from the native seeds about a healthy seed microbiome? A healthy seed microbiome (i) is diverse, rich, and evenly structured; (ii) contains bacteria, archaea, and fungi; (iii) contains microorganisms known for beneficial as well as for pathogenic interaction; and is (iv) highly specific. We found substantial differences to the microbiomes of crop seeds; the same has already been reported for the rhizosphere microbiome [19]. This is not surprising because cultivation pressure on seeds started around 8000 years ago and included, among others, the two major alterations on seed morphology: increased grain size and non-shattered seeds [55], where the latter predicated successful seed dispersal on human activity [56]. Today, seed treatments focus on uniform, clean, and pathogen-free seeds that are almost entirely produced commercially and traded globally [18]. In contrast, nature created a rich diversity of seed and fruit systems, with their own genotype-specific microbiomes. Figure 1 illustrates the morphological diversity of seeds and associated microbiota of the alpine plants investigated. Recent agriculture leads to a global landscape highly dominated by only few crop plants with desired characteristics. An enormous amount of 70% of wild relatives of modern crop plants are threatened with extinction [57]; consequentially, also their native microbiota with all their functional and metabolic skills are at risk of getting lost. For that reason, Berg and Raaijmakers [18] recently proposed international seed banks like Svalbard Global Seed Vault and Millennium Seed Bank to include conservation strategies for seed-associated microbiota. Based on the seed microbiota of wild ancestors or natural plants, microbial communities could be reconstructed with the ultimate goal to improve resilience of modern crops and reduce the amount of required pesticides.

## Conclusion

Undisturbed environments provide the best settings to explain indigenous plant-microbe interactions. Under such conditions, in a protected Alpine meadow, we found highly diversified and abundant seed microbiomes consisting of bacteria, archaea, and fungi. Moreover, despite growing together in the same soil, we found a higher degree of plant specificity than already discovered for crop seeds. All results underline the importance of plant-specific seed microbiota to ensure best-matching microbial symbionts for the next generation. However, network analysis captured consistent patterns of co-occurrence between bacteria and archaea in contrast to exclusion within the fungal community across all plant genotypes. This outlines the importance of cross-kingdom microbial interactions. We suggest that diversity associated with seeds may contribute to maintain soil microbial diversity, with importance for plasticity of the whole ecosystem. This knowledge can be translated into a better understanding of disease outbreaks and could be used for the production of resilient, healthy, and high-quality crop seeds.

## Additional files

Additional file 1: Abundance and composition of seed microbiota. **Table S1**: Mean microbial gene copy numbers in the seeds of different plant genotypes quantified by qPCR. **Figure S1**: Microbial composition of the seed core microbiomes of each plant genotype. (A) shows bacterial, (B) archaeal, and (C) fungal composition. Only bacteria and fungi occurring with at least 0.01% relative abundance in the whole dataset are shown. For low abundant archaea, no threshold was set to be included in the figure. Taxa not assigned to genus level are labeled with “sp.” after the lowest assignable taxonomic description. **Table S2**: Composition of the bacterial/archaeal microbiome with at least 0.1% abundance. **Table S3**: Composition of the fungal microbiome with at least 0.1% abundance. (DOCX 197 kb)

Additional file 2: Comparison of microbial diversity and composition between alpine seeds investigated. **Figure S2**: Comparison of bacterial and fungal diversity within alpine plant seeds. Shannon diversity indices were compared by grouping the samples according to their plant genotype (A), the life cycle of the plant (B), which is either annual or perennial, and the fruit type (C), either achene or capsule. Colors of the grouping variables are shown on either right. Calculated values and standard deviations can be looked up in Table S4. **Table S4**: Shannon diversity indices of seed samples grouped by plant genotype, life cycle and fruit type. **Table S5**: ANOSIM results of community composition dependency for bacteria and fungi on the three categorical variables. **Table S6**: Pairwise ANOSIM results comparing differences in bacterial and fungal community composition between the seeds of the eight plant genotypes. (DOCX 401 kb)

## Data Availability

The raw sequence data for the 16S rRNA gene and the ITS region are available from the European Nucleotide Archive (ENA) at study accession number PRJEB28955.
