# Supplementary material for: Seeds of native alpine plants host unique microbial communities embedded in cross-kingdom networks
Source: Microbiome. 2019 Jul 24;7:108. doi: 10.1186/s40168-019-0723-5 (PMC6651914; doi:10.1186/s40168-019-0723-5)
Supplement: Supplementary file 1 — Abundance and composition of seed microbiota. Table S1: Mean microbial gene copy numbers in the seeds of different plant genotypes quantified by qPCR. Figure S1: Microbial composition of the seed core microbiomes of each plant genotype. (A) shows bacterial, (B) archaeal, and (C) fungal composition. Only bacteria and fungi occurring with at least 0.01% relative abundance in the whole dataset are shown. For low abundant archaea, no threshold was set to be included in the figure. Taxa not assigned to genus level are labeled with “sp.” after the lowest assignable taxonomic description. Table S2: Composition of the bacterial/archaeal microbiome with at least 0.1% abundance. Table S3: Composition of the fungal microbiome with at least 0.1% abundance. (DOCX 197 kb) [file 40168_2019_723_MOESM1_ESM.docx]

**Additional file 1: Abundance and composition of seed microbiota.**

|  | Mean bacterial gene copy numbers/g seed | Mean archaeal gene copy numbers/g seed | Mean fungal gene copy numbers/g seed | Mean copy numbers of total microbiota/g seed |
| --- | --- | --- | --- | --- |
| *A. major* | 8.56E+10^a^ | 3.97E+08^a^ | 1.14E+11^ab^ | 2.00E+11^a^ |
| *E. rostkoviana* | 2.33E+11^ab^ | 2.29E+09^bc^ | 4.51E+11^bc^ | 6.87E+11^b^ |
| *G. asclepiadea* | 3.59E+11^bc^ | 1.13E+09^ab^ | 4.61E+11^bc^ | 8.21E+11^b^ |
| *G. germanica* | 5.20E+10^a^ | 2.94E+08^a^ | 6.39E+10^a^ | 1.16E+11^a^ |
| *H. quadrifida* | 1.78E+11^ab^ | 2.71E+09^c^ | 1.68E+11^ab^ | 3.48E+11^a^ |
| *P. palustris* | 5.30E+11^c^ | 4.75E+09^d^ | 6.97E+11^c^ | 1.22E+12^c^ |
| *R. glacialis* | 7.23E+11^d^ | 1.22E+10^e^ | 1.37E+12^d^ | 2.10E+12^d^ |
| *S. lucida* | 8.18E+10^a^ | 9.53E+08^a^ | 4.79E+10^a^ | 1.31E+11^a^ |

**Table S1:** Mean microbial gene copy numbers in the seeds of different plant genotypes quantified by qPCR.

Upper case letters indicate significant differences within microbial abundances where samples with same letters are not significantly different (p > 0.05) according to ANOVA including Tukey-HSD test correction.


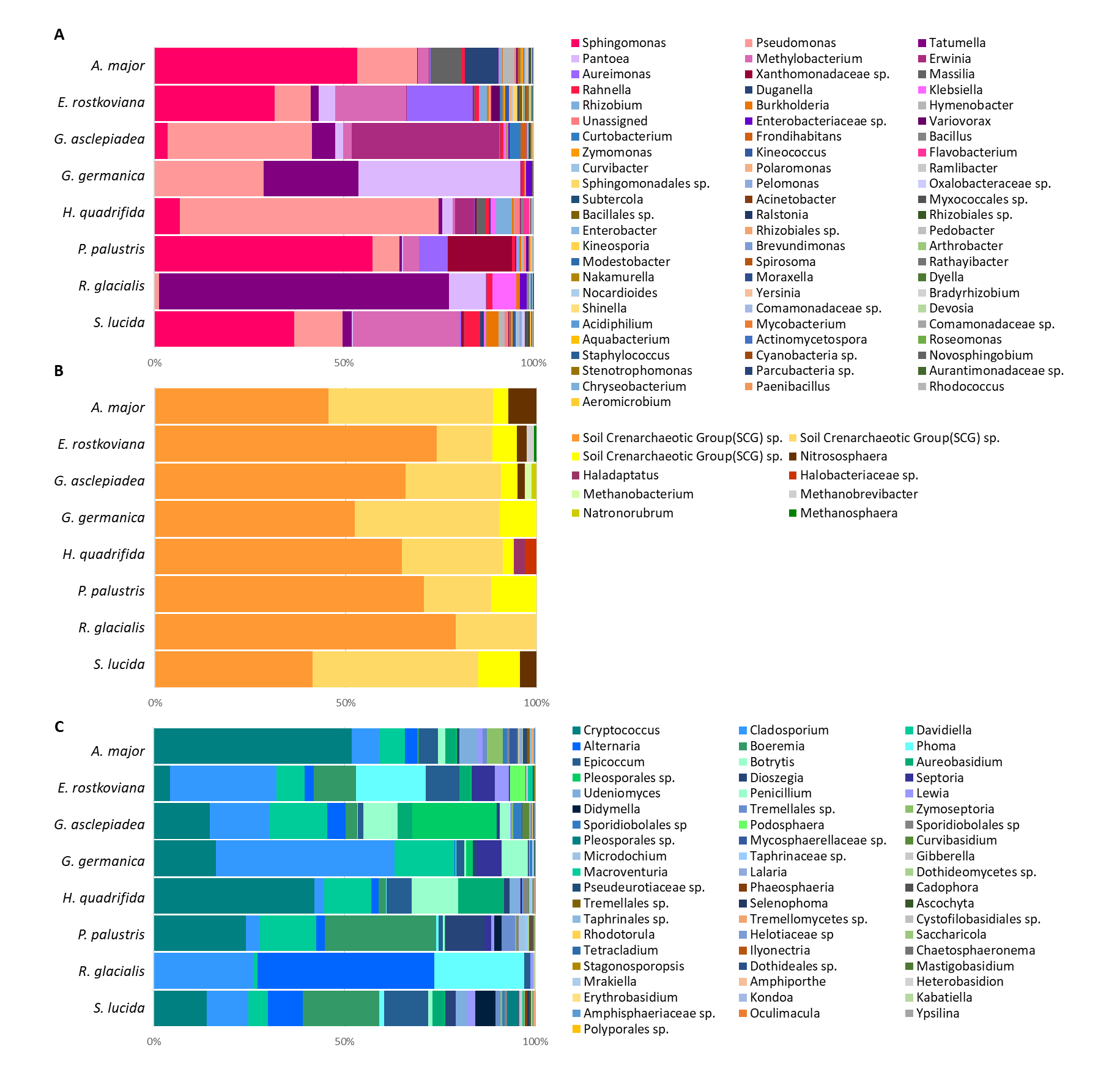


**Figure S1: Microbial composition of the seed core microbiomes of each plant genotype.** (A) shows bacterial, (B) archaeal and (C) fungal composition. Only bacteria and fungi occurring with at least 0.01% relative abundance in the whole dataset are shown. For low abundant archaea, no threshold was set to be included in the figure. Taxa not assigned to genus level are described by ‚sp.‘ after lower taxonomic description.

**Table S2:** Composition of the bacterial/archaeal microbiome with at least 0.1% abundance

| Family | Genus | Relative abundance in all samples (%) | Family | Genus | Relative abundance in all samples (%) |
| --- | --- | --- | --- | --- | --- |
| *Pseudomonadaceae* | *Pseudomonas* | 18.51 | *Phycisphaerae* | unidentified | 0.02 |
| *Sphingomonadaceae* | *Sphingomonas* | 18.47 | *Obscuribacterales* | unidentified | 0.02 |
| *Enterobacteriaceae* | *Tatumella* | 12.87 | *Opitutaceae* | *Opitutus* | 0.02 |
| *Methylobacteriaceae* | *Methylobacterium* | 5.84 | *Gaiellaceae* | *Gaiella* | 0.02 |
| *Enterobacteriaceae* | *Pantoea* | 4.71 | *Deinococcaceae* | *Deinococcus* | 0.02 |
| *Enterobacteriaceae* | *Erwinia* | 4.04 | *Acidobacteria* | unidentified | 0.02 |
| *unidentified* | *unidentified* | 3.98 | *Anaerolineaceae* | unidentified | 0.02 |
| *Anaplasmataceae* | *Wolbachia* | 3.82 | *Gemmatimonadaceae* | *Gemmatimonas* | 0.02 |
| *Enterobacteriaceae* | *Buchnera* | 2.41 | *Micrococcaceae* | *Micrococcus* | 0.02 |
| *Rhizobiaceae* | *Rhizobium* | 2.40 | *Xanthomonadaceae* | *Luteibacter* | 0.02 |
| *Aurantimonadaceae* | *Aureimonas* | 1.96 | *Roseiflexaceae* | *Roseiflexus* | 0.02 |
| *Enterobacteriaceae* | *Rahnella* | 1.50 | *Holophagae* | unidentified | 0.02 |
| *Oxalobacteraceae* | *Massilia* | 1.40 | *Soil Crenarchaeotic Group(SCG)* | unidentified | 0.02 |
| *Oxalobacteraceae* | *Duganella* | 1.31 | *Beijerinckiaceae* | unidentified | 0.02 |
| *Enterobacteriaceae* | *Klebsiella* | 1.30 | *Microbacteriaceae* | *Phycicola* | 0.02 |
| *Cytophagaceae* | *Hymenobacter* | 1.29 | *Acidobacteria* | unidentified | 0.01 |
| *Enterobacteriaceae* | unidentified | 1.24 | *Streptococcaceae* | *Streptococcus* | 0.01 |
| *Xanthomonadaceae* | unidentified | 1.16 | *Streptomycetaceae* | *Streptomyces* | 0.01 |
| *Microbacteriaceae* | *Curtobacterium* | 0.82 | *Simkaniaceae* | *Candidatus Rhabdochlamydia* | 0.01 |
| *Burkholderiaceae* | *Burkholderia* | 0.65 | *Armatimonadetes* | unidentified | 0.01 |
| *Microbacteriaceae* | *Frondihabitans* | 0.62 | *Enterobacteriaceae* | *Serratia* | 0.01 |
| *Oxalobacteraceae* | unidentified | 0.48 | *unidentified* | unidentified | 0.01 |
| *Sphingomonadaceae* | *Zymomonas* | 0.46 | *Corynebacteriaceae* | *Corynebacterium* | 0.01 |
| *Bacillaceae* | *Bacillus* | 0.41 | *Acidobacteria* | unidentified | 0.01 |
| *Comamonadaceae* | *Variovorax* | 0.37 | *Cytophagaceae* | *Fibrella* | 0.01 |
| *Kineosporiaceae* | *Kineococcus* | 0.25 | *unidentified* | unidentified | 0.01 |
| *Rhizobiales* | *unidentified* | 0.22 | *Microbacteriaceae* | *Amnibacterium* | 0.01 |
| *Myxococcales* | unidentified | 0.22 | *unidentified* | unidentified | 0.01 |
| *Comamonadaceae* | *Polaromonas* | 0.22 | *unidentified* | unidentified | 0.01 |
| *Enterobacteriaceae* | *Enterobacter* | 0.21 | *Comamonadaceae* | *Caenimonas* | 0.01 |
| *Moraxellaceae* | *Acinetobacter* | 0.21 | *Xiphinematobacteraceae* | *Xiphinematobacter* | 0.01 |
| *Microbacteriaceae* | *Subtercola* | 0.21 | *Acidimicrobiaceae* | uncultured | 0.01 |
| Other | unidentified | 0.20 | *Acetobacteraceae* | *Gluconobacter* | 0.01 |
| *Flavobacteriaceae* | *Flavobacterium* | 0.20 | *Cellulomonadaceae* | *Cellulomonas* | 0.01 |
| *Sphingobacteriaceae* | *Pedobacter* | 0.17 | *Chitinophagaceae* | *Chitinophaga* | 0.01 |
| *Comamonadaceae* | *Curvibacter* | 0.16 | *Nocardiaceae* | *Williamsia* | 0.01 |
| *Comamonadaceae* | *Aquabacterium* | 0.15 | *Coxiellaceae* | *Aquicella* | 0.01 |
| *Cytophagaceae* | *Spirosoma* | 0.15 | *Nitrosomonadaceae* | uncultured | 0.01 |
| *Comamonadaceae* | *Ramlibacter* | 0.14 | *Micromonosporaceae* | *Micromonospora* | 0.01 |
| *Acetobacteraceae* | *Acidiphilium* | 0.13 | *Comamonadaceae* | *Tepidimonas* | 0.01 |
| *Moraxellaceae* | *Moraxella* | 0.12 | *Planctomycetaceae* | *Isosphaera* | 0.01 |
| *unidentified* | unidentified | 0.11 | *Caulobacteraceae* | *Caulobacter* | 0.01 |
| *Enterobacteriaceae* | *Candidatus Hamiltonella* | 0.11 | *Micromonosporaceae* | *Luedemannella* | 0.01 |
| *Cyanobacteria* | *Cyanobacteria* | 0.11 | *Methylobacteriaceae* | *Microvirga* | 0.01 |
| *Paenibacillaceae* | *Paenibacillus* | 0.10 | *Rhodobacteraceae* | *Paracocccus* | 0.01 |
| *Nakamurellaceae* | *Nakamurella* | 0.09 | *Rhodospirillales Incertae Sedis* | *Candidatus Alysiosphaera* | 0.01 |
| *Comamonadaceae* | *Pelomonas* | 0.09 | *Nocardioidaceae* | *Marmoricola* | 0.01 |
| *Nocardioidaceae* | *Nocardioides* | 0.09 | *Acetobacteraceae* | unidentified | 0.01 |
| *Sanguibacteraceae* | *Sanguibacter* | 0.09 | *Acidothermaceae* | *Acidothermus* | 0.01 |
| *Mycobacteriaceae* | *Mycobacterium* | 0.08 | *Chthoniobacteraceae* | *Chthoniobacter* | 0.01 |
| *Hyphomicrobiaceae* | *Devosia* | 0.08 | *Bradyrhizobiaceae* | *Bosea* | 0.01 |
| *Caulobacteraceae* | *Brevundimonas* | 0.08 | *Planctomycetaceae* | *Gemmata* | 0.01 |
| *Burkholderiaceae* | *Ralstonia* | 0.08 | *Beijerinckiaceae* | *Methylorosula* | 0.01 |
| *Xanthomonadaceae* | *Stenotrophomonas* | 0.08 | *Flavobacteriaceae* | *Cloacibacterium* | 0.01 |
| *Kineosporiaceae* | *Kineosporia* | 0.08 | *Cellvibrionaceae* | *Cellvibrio* | 0.01 |
| *Comamonadaceae* | unidentified | 0.07 | *Geodermatophilaceae* | *Blastococcus* | 0.01 |
| *Geodermatophilaceae* | *Modestobacter* | 0.07 | *Neisseriaceae* | unidentified | 0.01 |
| *Sphingomonadaceae* | *Novosphingobium* | 0.07 | *Acidobacteria* | *Bryobacter* | 0.01 |
| *Parcubacteria* | unidentified | 0.07 | *Chitinophagaceae* | *Ferruginibacter* | 0.01 |
| *Bacillales* | unidentified | 0.07 | *Chloroflexi* | *unidentified* | 0.01 |
| *Gaiellales* | unidentified | 0.07 | *Cyanobacteria* | unidentified | 0.01 |
| *Cytophagaceae* | *Dyadobacter* | 0.06 | *Chitinophagaceae* | *Hydrotalea* | 0.01 |
| *Micrococcaceae* | *Arthrobacter* | 0.06 | *Chitinophagaceae* | unidentified | 0.01 |
| *Chthoniobacterales* | unidentified | 0.05 | *Intrasporangiaceae* | *Janibacter* | 0.01 |
| *Sphingobacteriaceae* | *Mucilaginibacter* | 0.05 | *Carnobacteriaceae* | *Atopostipes* | 0.01 |
| *Patulibacteraceae* | *Patulibacter* | 0.05 | *Comamonadaceae* | *Schlegelella* | 0.01 |
| *Acetobacteraceae* | *Roseomonas* | 0.05 | *Comamonadaceae* | *Acidovorax* | 0.01 |
| *Comamonadaceae* | unidentified | 0.05 | *Sphingobacteriaceae* | *Sphingobacterium* | 0.01 |
| *Microbacteriaceae* | *Rathayibacter* | 0.05 | *Microbacteriaceae* | unidentified | 0.01 |
| *Xanthomonadaceae* | *Dyella* | 0.05 | *Lactobacillaceae* | *Lactobacillus* | 0.01 |
| *Enterobacteriaceae* | *Candidatus Regiella* | 0.05 | *Alcaligenaceae* | *Achromobacter* | 0.01 |
| *Solirubrobacterales* | unidentified | 0.05 | *Clostridiaceae 1* | *Clostridium sensu stricto 13* | 0.01 |
| *Flavobacteriaceae* | *Chryseobacterium* | 0.05 | *Betaproteobacteria* | unidentified | 0.01 |
| *Acetobacteraceae* | unidentified | 0.05 | *Thiotrichaceae* | unidentified | 0.01 |
| *Micromonosporaceae* | *Actinoplanes* | 0.04 | *Kineosporiaceae* | *Quadrisphaera* | 0.01 |
| *Nocardiaceae* | *Rhodococcus* | 0.04 | *Xanthobacteraceae* | *Variibacter* | 0.01 |
| *Solirubrobacteraceae* | *Solirubrobacter* | 0.04 | *Actinobacteria* | unidentified | 0.01 |
| *Enterobacteriaceae* | *Yersinia* | 0.04 | *Latescibacteria* | unidentified | 0.01 |
| *Aurantimonadaceae* | unidentified | 0.04 | *Phyllobacteriaceae* | *Mesorhizobium* | 0.01 |
| *Bradyrhizobiaceae* | *Bradyrhizobium* | 0.04 | *Solirubrobacterales* | unidentified | 0.01 |
| *Enterobacteriaceae* | *Citrobacter* | 0.04 | *Hyphomicrobiaceae* | unidentified | 0.01 |
| *Blattabacteriaceae* | *Candidatus Brownia* | 0.03 | *Peptostreptococcaceae* | *Intestinibacter* | 0.01 |
| *Comamonadaceae* | *Delftia* | 0.03 | *Sphingomonadaceae* | *Sphingopyxis* | 0.01 |
| *Bdellovibrionaceae* | *Bdellovibrio* | 0.03 | *Armatimonadales* | unidentified | 0.01 |
| *Sphingomonadaceae* | *Polymorphobacter* | 0.03 | *Rhodospirillaceae* | *Defluviicoccus* | 0.01 |
| *Chloroflexi* | unidentified | 0.03 | *Legionellaceae* | *Legionella* | 0.01 |
| *Rhizobiaceae* | *Shinella* | 0.03 | *Cyanobacteria* | *Microcoleus* | 0.01 |
| *Planctomycetaceae* | uncultured | 0.03 | *Microbacteriaceae* | *Clavibacter* | 0.01 |
| *Planctomycetaceae* | *Singulisphaera* | 0.03 | *Actinobacteria* | unidentified | 0.01 |
| *Pseudonocardiaceae* | *Actinomycetospora* | 0.03 | *Nocardioidaceae* | unidentified | 0.01 |
| *Soil Crenarchaeotic Group(SCG)* | unidentified | 0.03 | *Chitinophagaceae* | *Terrimonas* | 0.01 |
| *Flavobacteriaceae* | *Empedobacter* | 0.03 | *Rhizobiales Incertae Sedis* | *Rhizomicrobium* | 0.01 |
| *Nocardioidaceae* | *Aeromicrobium* | 0.03 | *Planctomycetes* | unidentified | 0.01 |
| *Rhizobiaceae* | *Ensifer* | 0.03 | *Acidimicrobiaceae* | unidentified | 0.01 |
| *Thermomicrobia* | unidentified | 0.03 | *Rhizobiaceae* | *Neorhizobium* | 0.01 |
| *Flavobacteriaceae* | *Epilithonimonas* | 0.03 | *Acidobacteria* | *Candidatus Solibacter* | 0.01 |
| *Staphylococcaceae* | *Staphylococcus* | 0.02 | *Rhodobacteraceae* | *Amaricoccus* | 0.01 |
| *Acidobacteria* | unidentified | 0.02 | *Burkholderiales* | unidentified | 0.01 |
| *Pseudonocardiaceae* | *Pseudonocardia* | 0.02 | *Rickettsiales* | unidentified | 0.01 |
| Unknown Family | *Blastocatella* | 0.02 | *Spiroplasmataceae* | *Spiroplasma* | 0.01 |
| *Planococcaceae* | unidentified | 0.02 | *Rhodobiaceae* | unidentified | 0.01 |
| *Comamonadaceae* | *Comamonas* | 0.02 | *uncultured bacterium* | unidentified | 0.01 |
| *Sphingomonadaceae* | *Sphingobium* | 0.02 | *Polyangiaceae* | *Sorangium* | 0.01 |
| *Gemmatimonadaceae* | uncultured | 0.02 | *Planctomycetaceae* | *unidentified* | 0.01 |
| *Bradyrhizobiaceae* | *Tardiphaga* | 0.02 | *Xanthomonadaceae* | *Lysobacter* | 0.01 |
| *Frankiaceae* | *Jatrophihabitans* | 0.02 | *Iamiaceae* | *Iamia* | 0.01 |
| *Acidimicrobiales* | unidentified | 0.02 | *Enterobacteriaceae* | *Escherichia-Shigella* | 0.01 |
| *Oligoflexales* | unidentified | 0.02 | *Myxococcales* | unidentified | 0.01 |

**Table S3:** Composition of the fungal microbiome with at least 0.1% abundance

| Family | Genus | Relative abundance in all samples (%) | Family | Genus | Relative abundance in all samples (%) |
| --- | --- | --- | --- | --- | --- |
| *Tremellales* | *Cryptococcus* | 17.03 | *Phaeosphaeriaceae* | *Stagonospora* | 0.07 |
| *Ascomycota* | unidentified | 12.79 | *Helotiales* | *Oculimacula* | 0.06 |
| *Davidiellaceae* | *Davidiella* | 7.92 | *Dothideales* | unidentified | 0.05 |
| *Pleosporales* | *unidentified* | 7.75 | *Amphisphaeriaceae* | unidentified | 0.05 |
| *Pleosporaceae* | *Alternaria* | 6.84 | *Hypocreales* | *Myrothecium* | 0.04 |
| *Pleosporales* | *Phoma* | 5.53 | *Sporidiobolales* | *Sporobolomyces* | 0.04 |
| *Pleosporales* | *Boeremia* | 5.20 | *Erythrobasidiales* | *Erythrobasidium* | 0.04 |
| *Pleosporaceae* | *Epicoccum* | 3.02 | *Cystofilobasidiales* | *Mrakiella* | 0.04 |
| *Dothioraceae* | *Aureobasidium* | 2.72 | *Hypocreales* | *Ilyonectria* | 0.04 |
| *Sclerotiniaceae* | *Botrytis* | 2.49 | *Kondoaceae* | *Kondoa* | 0.04 |
| *Tremellales* | *Dioszegia* | 2.27 | *Spizellomycetaceae* | *Powellomyces* | 0.03 |
| *Cystofilobasidiaceae* | *Udeniomyces* | 2.14 | *Saccharomycetales* | *Debaryomyces* | 0.03 |
| *Trichocomaceae* | *Penicillium* | 2.00 | *Clavulinaceae* | *Clavulina* | 0.03 |
| *Erysiphaceae* | *Podosphaera* | 1.73 | *Lasiosphaeriaceae* | *Bagadiella* | 0.03 |
| *Pleosporales* | *Macroventuria* | 1.71 | *Lasiosphaeriaceae* | *Podospora* | 0.03 |
| *Pleosporaceae* | *Lewia* | 1.49 | *Ascomycota* | *Ypsilina* | 0.03 |
| *Pleosporales* | *Didymella* | 1.46 | *Parmeliaceae* | *Pseudevernia* | 0.03 |
| *Pleosporales* | *Mycocentrospora* | 1.14 | *Tremellales* | *Bullera* | 0.03 |
| *Mycosphaerellaceae* | *Septoria* | 1.01 | *Cystofilobasidiaceae* | *Cystofilobasidium* | 0.03 |
| *Helotiales* | *Cadophora* | 0.85 | *Davidiellaceae* | *Cladosporium* | 0.03 |
| *Sporidiobolales* | *unidentified* | 0.79 | *Ascomycota* | *Knufia* | 0.03 |
| *Sporidiobolales* | unidentified | 0.79 | *Nectriaceae* | *Flagellospora* | 0.03 |
| *Dothideomycetes* | *Zymoseptoria* | 0.76 | *Dothioraceae* | *Kabatiella* | 0.02 |
| *Tremellales* | *unidentified* | 0.75 | *Metschnikowiaceae* | *Metschnikowia* | 0.02 |
| *Phaeosphaeriaceae* | *Phaeosphaeria* | 0.71 | *Chaetothyriales* | unidentified | 0.02 |
| *Dothideomycetes* | unidentified | 0.68 | *Capnodiales* | unidentified | 0.02 |
| *Pleosporales* | unidentified | 0.54 | *Leucosporidiaceae* | *Leucosporidium* | 0.02 |
| *Mycosphaerellaceae* | unidentified | 0.43 | *unidentified* | unidentified | 0.02 |
| *Tremellales* | unidentified | 0.42 | *Helotiaceae* | *Hymenoscyphus* | 0.02 |
| *Nectriaceae* | *Gibberella* | 0.39 | *Cystofilobasidiaceae* | *Itersonilia* | 0.02 |
| *Pseudeurotiaceae* | unidentified | 0.38 | *Sordariomycetes* | unidentified | 0.01 |
| *Taphrinaceae* | unidentified | 0.38 | *Polyporales* | unidentified | 0.01 |
| *Microbotryomycetes* | *Curvibasidium* | 0.34 | *Leucosporidiaceae* | *Leucosporidiella* | 0.01 |
| *Pleosporales* | *Ascochyta* | 0.28 | *Phaeosphaeriaceae* | unidentified | 0.01 |
| *Taphrinaceae* | *Lalaria* | 0.26 | *Hypocreales* | *Acremonium* | 0.01 |
| *Helotiales* | *Chlorociboria* | 0.26 | *Tremellales* | *Tremella* | 0.01 |
| *Pleosporaceae* | *Pleospora* | 0.25 | *Ceratocystidaceae* | *Thielaviopsis* | 0.01 |
| *Dothioraceae* | *Selenophoma* | 0.25 | *Sporormiaceae* | *unidentified* | 0.01 |
| *Helotiaceae* | unidentified | 0.24 | *Sclerotiniaceae* | *Zoellneria* | 0.01 |
| *Pleosporales* | *Stagonosporopsis* | 0.22 | *Helotiales* | *Pilidium* | 0.01 |
| *Bondarzewiaceae* | *Heterobasidion* | 0.19 | *Clavicipitaceae* | *Claviceps* | 0.01 |
| *Taphrinales* | unidentified | 0.18 | *Mycosphaerellaceae* | *Cercospora* | 0.01 |
| *Xylariales* | *Microdochium* | 0.18 | *Saccharomycetales* | *Candida* | 0.01 |
| *Massarinaceae* | *Saccharicola* | 0.18 | *Botryobasidiaceae* | *Botryobasidium* | 0.01 |
| *Tremellomycetes* | unidentified | 0.18 | *Nectriaceae* | unidentified | 0.01 |
| *Glomerellaceae* | *Colletotrichum* | 0.15 | *Dothioraceae* | unidentified | 0.01 |
| *Leucosporidiaceae* | *Mastigobasidium* | 0.15 | *Hypocreales* | *Sarocladium* | 0.01 |
| *Cystofilobasidiales* | unidentified | 0.12 | *Helotiales* | *Helgardia* | 0.01 |
| *Helotiales* | *Tetracladium* | 0.12 | *Leptosphaeriaceae* | *Plenodomus* | 0.01 |
| *Sporidiobolales* | *Rhodotorula* | 0.12 | *Basidiomycota* | unidentified | 0.01 |
| *Ascomycota* | *Tumularia* | 0.11 | *Herpotrichiellaceae* | *Exophiala* | 0.01 |
| *Gnomoniaceae* | *Amphiporthe* | 0.10 | *Phaeosphaeriaceae* | *Ampelomyces* | 0.01 |
| *Ascomycota* | *Chaetosphaeronema* | 0.10 | *Diatrypaceae* | *Libertella* | 0.01 |
| *Taphrinaceae* | *Taphrina* | 0.09 | *Teratosphaeriaceae* | unidentified | 0.01 |
| *Helotiales* | unidentified | 0.09 | *Pleosporales* | *Periconia* | 0.01 |
| *Diaporthaceae* | *Diaporthe* | 0.09 | *Herpotrichiellaceae* | *Rhinocladiella* | 0.01 |
| *Agaricostilbaceae* | *Bensingtonia* | 0.09 | *Mytilinidiaceae* | *Lophium* | 0.01 |
| *Leptosphaeriaceae* | *Leptosphaeria* | 0.08 | *Lulworthiaceae* | *Zalerion* | 0.01 |
