# Supplementary material for: Seeds of native alpine plants host unique microbial communities embedded in cross-kingdom networks
Source: Microbiome. 2019 Jul 24;7:108. doi: 10.1186/s40168-019-0723-5 (PMC6651914; doi:10.1186/s40168-019-0723-5)
Supplement: Supplementary file 2 — Comparison of microbial diversity and composition between alpine seeds investigated. Figure S2: Comparison of bacterial and fungal diversity within alpine plant seeds. Shannon diversity indices were compared by grouping the samples according to their plant genotype (A), the life cycle of the plant (B), which is either annual or perennial, and the fruit type (C), either achene or capsule. Colors of the grouping variables are shown on either right. Calculated values and standard deviations can be looked up in Table S4. Table S4: Shannon diversity indices of seed samples grouped by plant genotype, life cycle and fruit type. Table S5: ANOSIM results of community composition dependency for bacteria and fungi on the three categorical variables. Table S6: Pairwise ANOSIM results comparing differences in bacterial and fungal community composition between the seeds of the eight plant genotypes. (DOCX 401 kb) [file 40168_2019_723_MOESM2_ESM.docx]

**Additional file 2: Comparison of microbial diversity and composition between alpine seeds investigated.**


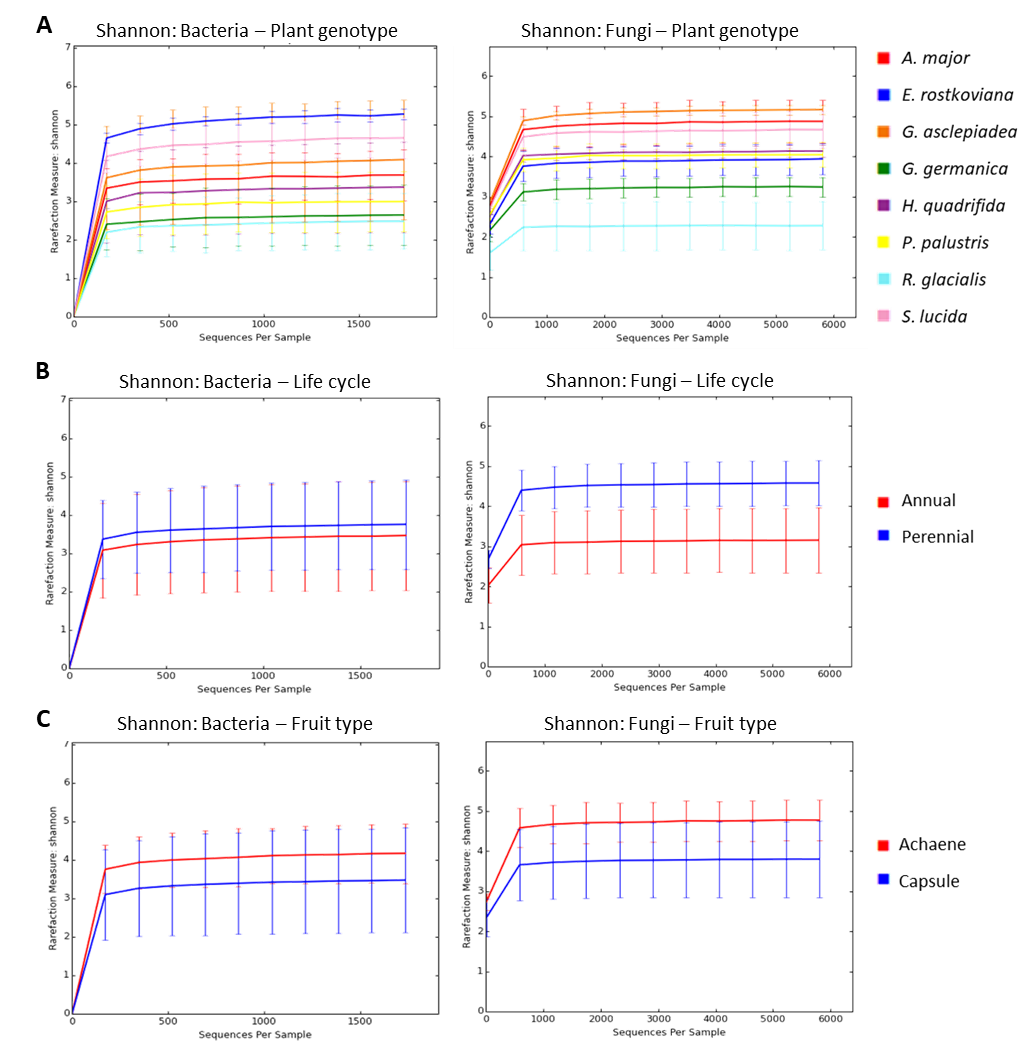


**Figure S2:** Comparison of bacterial and fungal diversity within alpine plant seeds. Shannon diversity indices were compared by grouping the samples according to their plant genotype (A), the life cycle of the plant (B), which is either annual or perennial and the fruit type (C), either achene or capsule. Colors of the grouping variables are shown on the either right. Calculated values and standard deviations can be looked up in Table S4.

**Table S4:** Shannon diversity indices of seed samples grouped by plant genotype, life cycle and fruit type.

**Table S5:** ANOSIM results of community composition dependency for bacteria and fungi on the three categorical variables.

* Dependency of the community composition was highly significant on genotype and life cycle for bacteria and fungi.

Table S6: Pairwise ANOSIM results comparing differences in bacterial and fungal community composition between the seeds of the eight plant genotypes.


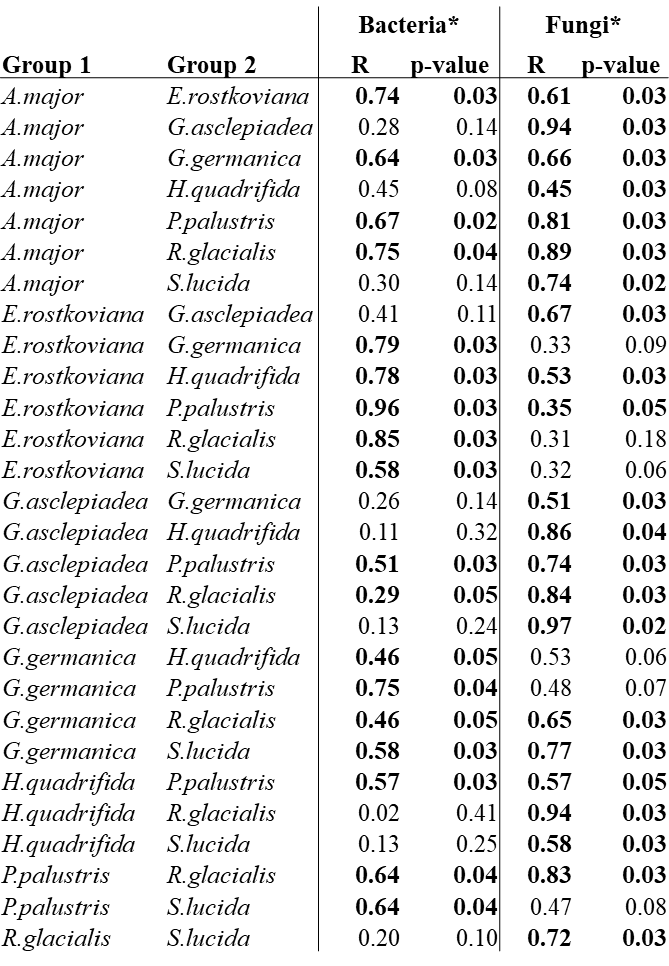


* Significant differences are highlighted in bold.
